# A low-fat spread with added plant sterols and fish omega-3 fatty acids lowers serum triglyceride and LDL-cholesterol concentrations in individuals with modest hypercholesterolaemia and hypertriglyceridaemia

**DOI:** 10.1007/s00394-018-1706-1

**Published:** 2018-05-03

**Authors:** Wendy A. M. Blom, Wieneke P. Koppenol, Harry Hiemstra, Tatjana Stojakovic, Hubert Scharnagl, Elke A. Trautwein

**Affiliations:** 1 Unilever Research and Development Vlaardingen, Olivier van Noortlaan 120, 3133 AT Vlaardingen, The Netherlands; 2 Clinical Institute of Medical and Chemical Laboratory Diagnostics, Medical University of Graz, Auenbruggerplatz 15, 8036 Graz, Austria; 3 PO Box 114, 3130 AC Vlaardingen, The Netherlands

**Keywords:** Plant sterols, Fish oil, DHA, EPA, Triglyceride, LDL-cholesterol, Human intervention

## Abstract

**Purpose:**

The primary and secondary objectives were to investigate the triglyceride (TG) and LDL-cholesterol (LDL-C) lowering effects of a spread with added plant sterols (PS) and fish oil as compared to a placebo spread.

**Methods:**

This study had a randomized, double-blind, placebo-controlled, parallel group design with two intervention arms. Following a 2-week placebo run-in period, 260 healthy individuals with modestly elevated blood TG (≥ 1.4 mmol/L) and LDL-C (≥ 3.4 mmol/L) concentrations consumed either the placebo or intervention spread for 4 weeks. The intervention spread contained 2.0 g/day PS and 1.0 g/day eicosapentaenoic acid (EPA) + docosahexanoic acid (DHA) from fish oil. Fasting serum lipids and apolipoproteins (Apo) (exploratory) were measured at the end of the run-in and intervention phases.

**Results:**

Four-week consumption of the intervention spread resulted in significantly lower TG (− 10.6%, 95% CI − 16.0 to − 4.9%; *P* < 0.001) and LDL-C concentrations (− 5.2%; 95% CI − 7.8 to − 2.4%) as compared to placebo. Total cholesterol (− 3.9%; 95% CI − 6.1 to − 1.5%), non-HDL-C (− 5.4%; 95% CI − 8.1 to − 2.7%), remnant-cholesterol (− 8.1%; 95% CI − 3.4 to − 12.5%), ApoAII (− 2.9%; 95% CI − 5.5 to − 0.2%), ApoCIII (− 7.7%; 95% CI − 12.1 to − 3.1%) and ApoB (− 3.2%; 95% CI − 5.9 to − 0.4%) concentrations were also significantly lower, as compared to placebo. No significant treatment effects were found for HDL-cholesterol, ApoAI, ApoCII, Apo E or ApoB/ApoAI.

**Conclusions:**

Four-week consumption of the intervention spread led to significant and clinically relevant decreases in serum TG, LDL-C and other blood lipid concentrations. The study was registered at clinicaltrials.gov (NCT 02728583).

**Electronic supplementary material:**

The online version of this article (10.1007/s00394-018-1706-1) contains supplementary material, which is available to authorized users.

## Introduction

Cardiovascular disease (CVD) is the leading cause of morbidity and mortality globally; 17.7 million of people die each year of CVD [1]. CVD includes numerous disorders. The most common types are cerebrovascular disease, coronary heart disease (CHD) and peripheral artery disease, many of which are related to atherosclerosis, i.e. the building up of plaque in the walls of the arteries. Elevated blood low-density lipoprotein-cholesterol (LDL-C) is the most important blood lipid risk factor of atherosclerosis [2, 3]. An emerging risk factor for the development of atherosclerosis are elevated blood triglycerides (TG).

Though an association of lowering TG and reduced CVD risk is still under debate, the evidence for a causal role of blood TG and specifically triglyceride-rich lipoproteins (TRL), i.e. chylomicron remnants, very-low-density lipoprotein (VLDL) and intermediate-density lipoprotein (IDL) in the development of atherosclerosis and CVD has steadily increased over the past years. The totality of data from observational studies, case control and angiography studies suggest that a modest association exists between elevated fasting TG concentrations and increased coronary heart disease (CHD) risk [4]. A meta-analysis of 29 prospective studies (*n* = 262,525) showed that per 1.0 mmol/L increase in fasting TG concentration, the risk of major CHD events increased by 27% [5]. Data from human genetic studies provide further evidence for a causal relationship between TGs and CHD risk [6]. Also, human intervention studies with fibrates and several meta-analyses of randomised controlled trials with lipid-modifying drugs provide further evidence that a lowering of fasting TG-levels reduces CHD risk, with the greatest benefit seen in individuals with hypertriglyceridemia [7–10]. A recent meta-analysis investigating the efficacy of TG-lowering drugs showed that TG-lowering drugs reduced risk of CVD events by 18% in subgroups with elevated TG and by 29% in individuals with dyslipidemia [i.e. high blood TG and low HDL-cholesterol (HDL-C)] while a benefit of CVD risk reduction per unit of TG-lowering was not reported [8].

The prevalence of elevated TG levels (≥ 1.7 mmol/L) is about 24% in the general US population (2009–2010 NHANES data) [11] and 36.7% of US adults aged ≥ 21 years were eligible for cholesterol treatment based on data collected between 2005 and 2012 [12]. Elevated blood TG concentration is a common feature in metabolic syndrome and insulin resistance. Many individuals at risk of Type 2 Diabetes Mellitus (T2DM) or having T2DM have dyslipidemia characterized by high blood TG and low HDL-C. NHANES data collected in 2009–2010 show that about 42% of people with T2DM has elevated TG concentrations and about 46% of people with T2DM have elevated LDL-C concentrations [13]. A study of primary care patients in Canada showed that among 134,074 patients who had completed a blood test for blood lipids, about 26% had elevated TG levels, about 26% had elevated LDL-C and 8% had a combination of elevated LDL-C and TG concentrations [14].

CVD is largely caused by diet and lifestyle-related factors. Early management of CVD risk factors by prevention and treatment of risk factors is important to reduce the lifetime risk of developing CVD. Life-long maintenance of normal LDL-C and normal TG levels may thus help to reduce the risk of developing CVD. Currently, guidelines for the prevention of CVD include promoting a healthy lifestyle through behaviour changes (i.e. healthy diet, physical activity, weight management and smoking cessation) and pharmacotherapy aimed to lower LDL-C and blood pressure [2, 3]. Guidelines of several international health organizations on the management and treatment of dyslipidemia acknowledge the importance of diet and lifestyle in CVD prevention [15, 16]. These guidelines also address the potential benefits of dietary adjuncts such as plant sterols (2 g/day) and high-dose (2–4 g/day) marine omega-3 fatty acids in the treatment of dyslipidemia.

PS naturally occur in all foods of plant origin and are found in everyday foods like vegetable oils, nuts, seeds, grain products, fruits and vegetables. The average daily intake of PS is approximately 200–300 mg/day with a typical Western European diet [17, 18] and about 300 to 450 mg/day with a vegetarian or vegan diet [19]. Consumption of PS at levels of 1.5 to 3.0 g/day significantly lowers blood LDL-C concentrations and also to some extent reduces TG concentrations, especially in those individuals with elevated basal TG [20, 21].

EPA and DHA are very long chain omega-3 fatty acids that are found in (fatty) fish. Most global dietary guidelines recommend a daily intake of EPA + DHA of at least 250 mg/day [22], but most people do not meet these recommendations [23]. Pharmacological doses of EPA + DHA prescribed to patients with hypertriglyceridemia are in the order of 2–4 g/day of EPA + DHA.

In this study, we investigated, in a population of healthy individuals with elevated LDL-C and borderline-high to high TG concentrations, the efficacy of a dietary approach that may help to lower both TG and LDL-C concentrations. We developed a spread with 2.0 g/day added PS and 1.0 g/day EPA + DHA from fish oil and tested whether this intervention spread would lower TG concentrations (primary objective) in addition to LDL-C and other blood lipids (secondary objective) as compared to a placebo spread. Apolipoprotein measures were included to explore if effects on TG and other blood lipid concentrations were reflected by changes in apolipoprotein concentrations.

The doses of EPA + DHA (1.0 g/day) and PS (2.0 g/day) were selected based on the results of a previous study in which we investigated the dose–response relationship between several low doses (< 2 g/day) of EPA + DHA in combination with 2.5 g/day of plant sterols, and TG concentrations [24].

## Subjects and methods

### Study population

The study was conducted at Charité Research Organisation (Berlin, Germany) in men and women aged between 18 and 75 years. Study participants were recruited by advertisements in local newspapers and on the internet. Individuals were included when they had fasting TG concentrations at screening ≥ 1.40 and ≤ 5.60 mmol/L and fasting LDL-C concentrations at screening ≥ 3.4 and ≤ 4.9 mmol/L. Study participants had to habitually consume spreads or be willing to consume spreads. Those with a recently (< 6 months) diagnosed cardiovascular event or medical history that might impact study measurements were excluded from participation. Other exclusion criteria were alcohol abuse, smoking, intense sporting activities (> 10 h/week), night shift work 2 weeks prior to screening or during the study, the use of oral antibiotics or over-the-counter and prescribed medication which may interfere with study outcomes and consumption of plant-sterol/stanol-enriched foods or supplements, fish oil and/or EPA or DHA supplements. The use of low and moderate doses of statins was allowed if dose and brand were stable for ≥ 3 months. Pregnant or lactating women were excluded from participations, as well as individuals with reported weight loss or gain of 3 kg or more during a period of 2 months prior to screening. Employees of Unilever or Charité Research Organisation were not eligible for participation, neither were individuals who donated blood 1 month (men) or 2 months (females) prior to screening, or who reported participation in another nutritional or biomedical trial 2 months prior to screening or during the study.

### Study design

This study was designed as a randomized, double-blind, placebo-controlled, parallel group study with two intervention arms. All study participants followed a 2-week run-in period during which they consumed the placebo spread to get familiarized with consuming a spread product and to allow blood lipid concentrations to stabilize. After the run-in phase, study participants were randomly allocated to consume either the placebo or the intervention spread for 4 weeks. Placebo and intervention spreads were provided in 12.5 g portion packs. Study participants were instructed to consume two portion packs daily divided over two main meals, i.e. breakfast, lunch or dinner.

At the end of the run-in and the intervention phases, fasting blood samples were collected on two consecutive days for measuring serum lipids (based on double blood sampling). Apolipoprotein concentrations were measured in single blood samples taken at the end of the run-in and the intervention phases.

### Study products and dietary restrictions

The study products consisted of low-fat spread with no added PS or fish oil (placebo product) and a low-fat spread with 2.0 g PS (dose expressed as free PS equivalents) added in the form of PS esters and 1.0 g EPA + DHA from fish oil (intervention product) formulated in 25 g of product. The PS esters (BASF Corporation) consisted of 60% PS and 40% fatty acid esters. The long-chain omega-3 fatty acids EPA and DHA were obtained from marine fish oil (MEG-3™, DSM, Basel, Switzerland). The EPA + DHA content of the commercial fish oil used for this study was about 30%, with a ratio of EPA to DHA of about 1:1. The composition of the study products is provided in Table 1. Part of the sunflower oil present in the placebo product was replaced by PS esters and fish oil in the intervention product. The rest of the formulation was kept similar. Due to this difference in formulation, total fat content and composition differed slightly between the two products. All study products were produced at Unilever Research and Development Vlaardingen, the Netherlands. Concentration of PS and EPA/DHA were measured in a random selection of the study products to check correct production of the products.

**Table 1:** Study product composition

Per 25 g product	Placebo	Intervention
Energy, kJ	393.0	319.1
Total protein (g)	0.0	0.0
Total carbohydrates (g)	0.0	0.0
Total fat (g)	10.6	8.6
SAFAs (g)	2.2	2.8
MUFAs (g)	2.6	2.0
PUFAs (g)	5.8	3.7
LA (g)	5.7	2.1
ALA (g)	0.0	0.0
EPA (mg)	0	543
DHA (mg)	0	460
TFAs (g)	0.1	0.1
Cholesterol (mg)	0.0	39.9
PS ester (g)^a^	0	3.3
Sodium (mg)	2.7	2.7
Vitamin E (mg)	7.5	3.5
Water (g)	14.3	14.3

^a^3.3 g of PS esters equals to 2.0 g of free PS

Some study products were administered at the test facility, i.e. when study participants were provided with breakfast, after collection of fasting blood samples. Study participants self-administered the rest of the study products (during run-in and intervention) at home. They were supplied with study products, in cooling bags, at the first day of the run-in period (Day − 14) for the next 2 weeks and at the first day of the intervention period (Day 1) for the next 4 weeks. Last product consumption was on Day 28. Spare products were provided in case of loss of products. Study participants were requested to consume the spread on bread or on crackers; using the spread on top of hot dishes or cooking, baking or frying with the spreads was not allowed. They were asked to refrigerate the study products at home (1–7 °C) while freezing of study products was not allowed. Unused portion packs were returned to the study site. Study participants received instructions to minimize changes in their habitual diet and lifestyle during the entire study period and to refrain from consuming fish oil (EPA and DHA) supplements or foods enriched with PS or plant stanol esters during the study. They were also asked to consume a maximum of one serving of fish per week. A personal diary which clearly described restrictions and in which non-compliance could be written down was provided. Compliance with study product intake and dietary restrictions, use of concomitant medication and adverse events were monitored throughout the study.

### Blood sampling and assays

Venous blood samples were collected after an overnight fast (at least 10 h) at the last day of the run-in period (Day − 1), and at the first day of the intervention period (Day 1) and at two separate days at the end of the intervention period (Day 28 and Day 29).

All blood lipids, i.e. TC, HDL-C, LDL-C and TG were analysed by SYNLAB pharma institute, a division of SYNLAB Umweltinstitut GmbH, Berlin, Germany, using validated colourimetric methods on an AU680 automated analyser (Beckman Coulter, Beckman Coulter GmbH, Krefeld, Germany). All reagents and calibration standards were obtained from Beckman Coulter. The Root Mean Squared Deviation (between day) was ≤ 4.2% for the entire study period. Non-HDL-C (TC minus HDL-C) and remnant cholesterol (remnant C) defined as TC minus HDL-C and LDL-C (for post hoc analysis) concentrations were calculated.

Apolipoproteins (ApoAI, ApoAII, ApoB, ApoCII, ApoCIII and ApoE) were analysed at the Clinical Institute of Medical and Chemical Laboratory Diagnostics at the Medical University of Graz, Austria, in samples collected at Day 1 and Day 29 using commercially available assays and reagents. Apolipoproteins were determined by immunoturbidimetry using reagents from DiaSys (Holzheim, Germany) and standards from Siemens (Marburg, Germany, apoAI, apoB, apoE) and Kamiya Biomedical (Seattle, WA, USA, apoAII, apoCII, apoCIII). All measurements were performed on an Olympus AU640 automatic analyzer (Beckman Coulter, Brea, CA, USA). The coefficients of variation (between day) were < 5%. The ApoB/ApoAI ratio was calculated.

### Statistical analyses

The study was powered to detect an 8.2% lower TG concentration at the end of intervention for the intervention group as compared to the placebo group, with a power of 0.8 and a significance level alpha of 0.05 (two-sided). Study size calculations were based on simulations due to a skewed truncated distribution of TGs (1.4 to 5.7 mmol/l). The required sample size was calculated to be 260 (130 in each arm), considering a drop-out rate of 10%.

Data were analysed for the intent-to-treat (ITT) (i.e. all randomized study participants) and per-protocol (PP) populations, excluding for the PP analysis data of individuals who had been noncompliant with the study protocol. Here we only report data from the ITT population; the PP analysis yielded similar results (see Online Resource I).

Blood lipid concentrations determined on two consecutive days before and after intervention were averaged. All averaged blood lipid concentration (baseline as well as post-intervention) were subsequently log-transformed. The effects of intervention on blood lipids and apolipoproteins were analysed with an ANCOVA model using change from baseline on a log-scale as the response. Gender, baseline, age, weight, waist circumference, use of statins and the interaction-terms between treatment and gender and statin use were included in the model as covariates but were dropped from the model if they did not contribute to the model based on the Bayesian Information Criterion (BIC) as a goodness of fit criterion.

A two-sided significance level of alpha = 0.05 was used. Treatment effects were reported as an estimate of the relative change in blood lipids (expressed as a percentage and an associated 95% confidence interval) using the placebo treatment as a reference. LSmeans of blood lipids at the end of intervention (mean Day 28 and Day 29) are reported after back transformation with confidence intervals for each treatment group. All analyses were performed with the statistical software package SAS version 9.4 (SAS Institute Inc., Cary, NC, USA).

## Results

### Subject characteristics and compliance

A total of 1014 individuals were screened of which 265 were included in the run-in period. Five individuals were excluded from randomization due to non-compliance and were replaced. A total of 260 individuals were randomized into the study, of whom 259 completed the study (1 drop-out). Seven study participants were deemed noncompliant with the study protocol. These seven participants were excluded from the PP population but included in the ITT population. A subject flow diagram is provided in Online Resource II. Compliance to study product intake was excellent. A total of 238 out of 259 study participants had a 100% study product compliance during the intervention period. The other 21 study participants had a study product compliance > 90%. Study product compliance did not differ between intervention and placebo. Study products were well tolerated. Baseline characteristics of the randomized study participants are given in Table 2. Baseline characteristics did not significantly differ between the two groups.

**Table 2:** Subject characteristics at baseline (start of intervention)

	Placebo group (*n* = 130)	Intervention group (*n* = 130)
Men/women (*n*)	77/53	81/49
Age (years)	51.0 ± 10.0	51.6 ± 11.5
Body weight (kg)	87.3 ± 15.9	84.9 ± 15.4
Height (cm)	174.1 ± 9.9	174.4 ± 9.4
BMI (kg/m^2^)	28.8 ± 4.6	27.8 ± 4.1
Waist circumference (cm)		
Men	99.3 ± 8.4	100.2 ± 8.4
Women	92.9 ± 11.4	87.6 ± 11.1
TG (mmol/L)	1.99 ± 0.84	1.93 ± 1.05
TC (mmol/L)	6.13 ± 0.84	6.05 ± 0.85
LDL-C (mmol/L)	4.24 ± 0.64	4.16 ± 0.58
HDL-C (mmol/L)	1.22 ± 0.27	1.24 ± 0.29

Data represent mean ± SD

Baseline characteristics did not differ statistically significant (*p* > 0.05) between treatments

*BMI* body mass index, *TG* triglyceride, *LDL-C* low-density lipoprotein cholesterol, *HDL-C* high-density lipoprotein cholesterol, *TC* total cholesterol

### Serum lipids

After 4 weeks consumption of the spread with added PS and fish oil, serum TG concentrations were 10.6% (95% CI − 16.0 to − 4.9%; *p* < 0.001) lower as compared to placebo. Also, serum concentrations of LDL-C (− 5.2%; 95% CI − 7.8 to − 2.4%), TC (− 3.9%; 95% CI − 6.1 to − 1.5%), non-HDL-C (− 5.4%; 95% CI − 8.1 to − 2.7%) and remnant-C (− 8.1%; 95% CI − 3.4 to − 12.5%) were significantly (*p* ≤ 0.001) lower as compared to placebo (see Table 3). HDL-C did not differ between the two treatment groups (1.3; 95% CI − 0.7 to 3.3%; *p* = 0.220).

**Table 3:** Effects of placebo and intervention treatments on blood lipid and apolipoprotein measures

Outcome parameter	Placebo group End-of-intervention (LS means + 95% CI)	Intervention group End-of-intervention (LS means + 95% CI)	Absolute difference in LSMeans vs Placebo (95% CI)	Relative difference in LSMeans vs Placebo (95% CI)	*p* value
	mmol/L	mmol/L	mmol/L	%	
TG (mmol/L)	1.85 (1.77 to 1.93)	1.65 (1.58 to 1.73)	− 0.20 (− 0.31 to − 0.09)	− 10.6 (− 16.0 to − 4.9)	< 0.001
LDL-C (mmol/L)	4.22 (4.13 to 4.30)	4.00 (3.92 to 4.08)	− 0.22 (− 0.33 to − 0.10)	− 5.2 (− 7.8 to − 2.4)	< 0.001
TC (mmol/L)	6.04 (5.94 to 6.15)	5.81 (5.71 to 5.91)	− 0.23 (− 0.38 to − 0.09)	− 3.9 (− 6.1 to − 1.5)	0.001
HDL-C (mmol/L)	1.25 (1.23 to 1.26)	1.26 (1.24 to 1.28)	0.02 (− 0.01 to 0.04)	1.3 (− 0.7 to 3.3)	0.220
Non-HDL-C (mmol/L)	4.77 (4.67 to 4.87)	4.51 (4.42 to 4.60)	− 0.26 (− 0.31 to − 0.12)	− 5.4 (− 8.1 to − 2.7)	< 0.001
Remnant-C (mmol/L)	0.54 (0.52 to 0.56)	0.49 (0.48 to 0.51)	− 0.04 (− 0.07 to − 0.02)	− 8.1 (− 3.4 to − 12.5)	0.001
ApoAI (mg/dL)	135.3 (133.6 to 137.0)	135.1 (133.4 to 136.8)	− 0.14 (− 2.53 to 2.25)	− 0.1 (− 1.8 to 1.7)	0.905
ApoAII (mg/dL)	40.1 (39.3 to 40.8)	38.9 (38.1 to 39.6)	− 1.17 (− 2.24 to − 0.10)	− 2.9 (− 5.5 to − 0.2)	0.033
ApoCII (mg/dL)	5.4 (5.2 to 5.6)	5.2 (5.0 to 5.4)	− 0.17 (− 0.43 to 0.09)	− 3.2 (− 7.8 to 1.6)	0.190
ApoCIII (mg/dL)	13.9 (13.4 to 14.4)	12.8 (12.4 to 13.3)	− 1.08 (− 1.73 to − 0.43)	− 7.7 (− 12.1 to − 3.1)	0.001
ApoE (mg/dL)	12.1 (11.8 to 12.4)	12.1 (11.8 to 12.4)	0.02 (− 0.40 to 0.44)	0.2 (− 3.2 to 3.7)	0.923
ApoB (mg/dL)	99.7 (97.7 to 101.8)	96.5 (94.6 to 98.5)	− 3.18 (− 5.99 to − 0.37)	− 3.2 (− 5.9 to − 0.4)	0.027
ApoB/ApoAI (mg/dL)	0.74 (0.72 to 0.75)	0.72 (0.70 to 0.73)	− 0.02 (− 0.04 to 0.00)	− 2.7 (− 5.5 to 0.3)	0.075

*TG* triglyceride, *LDL-C* low-density lipoprotein cholesterol, *HDL-C* high-density lipoprotein cholesterol, *TC* total cholesterol, *apo* apolipoprotein, *LS* least square

### Apolipoproteins

The results of the apolipoprotein analyses are also presented in Table 3. In short, the intervention resulted in significantly lower concentrations of ApoAII (− 2.9%; 95% CI − 5.5 to − 0.2%), ApoCIII (− 7.7%; 95% CI − 12.1 to − 3.1%) and ApoB (− 3.2%; 95% CI − 5.9 to − 0.4%) concentrations as compared to placebo. There were no statistically significant treatment effects on ApoAI, ApoCII, Apo E or ApoB/ApoAI.

### Adverse events

A total of 67 adverse events (AEs) were reported in 56 study participants, of which 30 were reported in the group that received the intervention product. Most AEs were not related to study product and/or procedures, only seven of the AEs were unlikely related, and one AE was judged by the investigator as possibly treatment related, i.e. diarrhoea on one morning during the placebo run-in phase. Most common reported AEs were headache and common cold.

## Discussion

This intervention study showed that consumption of a low-fat spread delivering 1.0 g/day of EPA and DHA from fish oil together with 2.0 g/day of PS significantly lowered serum TG as well as serum LDL-C, TC, non-HDL-C and remnant-C concentrations, as compared to placebo, in a population with elevated LDL-C and borderline-high to high TG concentrations.

Our findings confirm that consumption of a spread containing a recommended dose of PS and low doses of EPA + DHA can help lower both TG and LDL-C concentrations confirming that this dietary approach can contribute to maintaining low TG and LDL-C concentrations and thus to reducing the risk of developing CVD.

So far, seven previous clinical studies investigated the blood lipid-lowering effects of a combination of PS and EPA + DHA using different food formats [24–30]. One of these studies, performed by our group [24], using a similar low-fat spread format, investigated the dose–response relation for the TG-lowering effect of different doses of EPA/DHA in combination with a fixed dose of PS. That study showed that consumption of 0.9, 1.3 and 1.8 g/day of EPA + DHA in combination with 2.5 g of PS lowered TG concentrations by 9.3, 13.9, and 16.2%, respectively [24]. Our current finding of a 10.6% reduction in TG concentrations with 1.0 g EPA/DHA and 2.0 g PS is in line with these previous findings. Three other groups also investigated the effects of low-dose EPA/DHA in combination with PS [25, 28, 29]. Micallef and Garg [25] studied the effects of 3-week consumption of either sunola oil or EPA/DHA (1.4 g/day) capsules alone or in combination with a spread containing 2 g of PS per day in a hyperlipidemic study population. The combination of EPA/DHA and PS reduced LDL-C by 12.5% and TG by 25.9% as compared to baseline. Sunola oil had no significant effect on blood lipids. Bitzur et al. also observed significant (19%) lowering of blood TG after 12-week consumption of capsules containing 1.6 g PS and 1.3 g EPA/DHA per day, but the LDL-C lowering effect observed after 6 weeks treatment (− 5.9%) was not sustained at 12 weeks [29]. AbuMweis et al. failed to show in their study a blood lipid benefit after 4-week consumption of a margarine containing a combination of PS (22 mg/bw; mean: 1.7 g/day; range 1.0–1.8 g/day) and EPA/DHA (mean 1.1 g/day, range 0.7–2.1) or PS alone [28]. They argued that the lack of effect on LDL-C may be the result of the frequency and timing of product consumption as a single dose was provided with breakfast. Other studies testing PS (1.7–1.9 g/day) in combination with higher (2.0 and 5.4 g/day) doses of EPA/DHA showed significant reductions in TG concentrations and small or no reductions in LDL-C [26, 27, 30]. These studies also delivered the treatment as a single dose at breakfast, provided as oil or formulated in a yogurt drink. A meta-analysis of randomised controlled trials investigating the LDL-C lowering effects of PS and plant stanols indeed suggested that single intakes seem less efficacious in reducing LDL-C as compared to multiple daily intakes [31]. Unpublished data from subgroup analyses performed for another meta-analysis [21] also suggest lower efficacy with single vs. multiple daily intakes. This analysis also highlighted the importance of the time of intake with once a day consumption of PS with breakfast seeming to be less efficacious than with lunch or dinner. In our study, products were divided over at least two main meals (breakfast, lunch or dinner) which may have contributed to the observed efficacy.

Nevertheless, the LDL-C lowering effect of 5.2% (− 7.8 to − 2.4) observed in our study appears to be on the lower end of what is expected for a 2 g/day PS intake. The most comprehensive meta-analysis of PS intervention studies suggests an average LDL-C lowering of 7.6% (− 8.6 to − 6.7) with a dose of ≥ 1.5–< 2.0 g/day and an average LDL-C lowering of 8.0% (− 7.0 to − 9.0) with a dose of ≥ 2.0–< 2.5 g/ [21]. Possible factors that might have attenuated the magnitude of the LDL-cholesterol-lowering effects of PS include possible effects of DHA on LDL-C and the higher saturated fat content of the intervention spread versus placebo spread. In contrast to EPA, which has a neutral effect on LDL-C, DHA has been shown to modestly increase LDL-C concentrations [32]. A pooled analysis of six studies directly comparing EPA and DHA showed that DHA increased LDL-C by 0.12 mmol/L as compared to EPA [32]. Doses of DHA in these studies ranged between 2.3 and 4.0 g/day, which is however much higher than the DHA content of the spread used in our current study. Our spreads contained 1.0 g of EPA + DHA per 25 g daily serving, of which DHA contributed about 0.5 g. Effects of DHA on LDL-C concentrations are therefore expected to be small. Results of our previous dose–response study also showed that increasing dosages of EPA + DHA up to 1.8 g/day did not affect LDL-C concentration [24]. It is thus unlikely that the fish oil content of the spreads has weakened the effect of PS on LDL-C.

In our study, fish oil replaced sunflower oil in the intervention spread which impacted the fat composition of this product. Consequently, the intervention spread had a slightly higher saturated fat (SAFA) and cholesterol content, and less unsaturated fats, especially polyunsaturated fat (PUFA), as compared to the placebo product. This could have possibly affected blood lipid outcomes. Using equations from a recent systematic review and regression analysis looking at the effects of SAFA on serum lipids and lipoproteins [33], we predicted how the difference in fat content and composition between the two spreads could have affected LDL-C and TG concentrations. The results indicate that the fatty acid composition of the placebo spread would result in 0.03 mmol/L lower LDL-C and 0.02 mmol/L lower TG concentrations as compared to the intervention spread, which accounts for about 0.7 and 0.9% of baseline values, respectively. Hence, the differences in fat content and composition of the spreads may have slightly dampened the blood lipid lowering effects of the intervention.

The vitamin E content also differed between the intervention and placebo spreads, but it is unlikely that this affected the blood lipid results as vitamin E is not known to have a substantiated effect on blood lipid concentrations [34].

The TG-lowering effect of EPA + DHA from fish oil is well known. Based on a recent meta-analysis, consumption of 1.0 g of EPA/DHA per day is estimated to result in a TG-lowering of at least 0.162 mmol/L, which would account for a 8% reduction in our study population with baseline TG concentrations of about 2.0 mmol/L [35]. However, it is likely that also PS contributed to the TG-lowering effect observed in our study [20, 36]. Data from a pooled analysis by Demonty et al. [20] showed that PS intake at doses of 1.6 to 2.5 g/day lowered serum TG by on average 6.0% with more pronounced effects in individuals with higher baseline TG concentrations.

Regarding mechanism(s) of action, animal studies suggest that PS may lower TGs via interference with fatty acid absorption within the intestinal lumen, modulation of hepatic de novo lipogenesis and reduction in circulating medium and large VLDL particles [36]. The main mechanisms by which EPA and DHA lower blood TGs concentrations are inhibition of hepatic TG synthesis, decreased VLDL synthesis and secretion, and increased plasma lipoprotein lipase (LPL) activity [37–39]. Since PS and fish oil seem to lower TG concentrations partly via the same mechanisms, it seems unlikely that their effects would be fully additive.

To get more insights into the effects of PS and EPA + DHA on the underlying lipoprotein metabolism, we calculated remnant-C (cholesterol content of the remnant particles or TRLs) and non-HDL-C (and measured several apolipoproteins), next to LDL-C and TG concentrations. We observed that combined intake of PS and EPA/DHA in this study was accompanied by decreases in non-HDL-C and remnant-C, suggesting that next to a reduction in LDL-C also the cholesterol content of the TRLs decreased. This is in line with results of our previous study in which a lipoprotein analysis confirmed that the combination of PS and fish oil dose-dependently reduced VLDL-cholesterol and VLDL-TG [40]. The observed decrease in apoB concentrations suggests that the total number of pro-atherogenic particles (i.e. non-HDL lipoproteins) was reduced, which could reflect inhibition of VLDL production. We also observed a decrease in apoCIII concentrations, an important regulator of TG metabolism, which is expected to result in less inhibition of LPL, and hence increased lipolysis of TGs and lower circulating TG concentrations [41]. No effects of treatment on ApoAI concentrations were observed, which is in line with the HDL-C data.

The relevance of TG-lowering for CVD prevention has been shown in randomised controlled trials (RCTs) that tested TG-lowering drugs such as fibrates [8]. Fibrates are mainly prescribed in combination with statins, to high-risk patients with elevated TG, because the efficacy of fibrates on top of statins on CVD outcomes seems most evident in this population [16]. Whether TG-lowering via omega-3 fatty acids also lowers risk of CVD needs to be established.

Observational studies have consistently shown that fish consumption has a clear benefit for primary prevention of CVD [42]. However, recent RCTs investigating the effect of supplementation with the marine omega-3 fatty acids EPA + DHA on development of CVD, do not show benefits in secondary prevention of CVD [43–45]. Several reasons for this have been postulated, i.e. relatively short-term duration of omega-3 fatty acid intake, relatively low doses of EPA + DHA used, or masking of efficacy by concurrent effective drug therapies. In addition, none of the RCTS were specifically designed to target patients with elevated blood triglycerides. Two large RCTs with higher doses of omega-3 fatty acids are currently ongoing (REDUCE-IT, STRENGHT). These RCTs also included subjects with hypertriglyceridemia and will hopefully provide more definite answers about the potential role of marine omega-3 fatty acids in CVD prevention.

The AHA recently published a Science Advisory document on omega-3 PUFA and the prevention of clinical CVD based on a review of large RCTs with major clinical CVD end points [46]. Based on this, the AHA still recommends treatment with omega-3 fatty acid supplements for secondary prevention of CHD and sudden cardiac death among patients with prevalent CHD, and for secondary prevention of outcomes in patients with heart failure. For other populations or indications either no recommendation is provided (due to lack of studies) or treatment with omega-3 fatty acids is not indicated (due to lack of benefit).

The major strength of the current study was the randomized, double-blind, placebo-controlled study design and with 260 the large number of participants included in this study, making this one of the largest intervention studies investigating the combined effect of PS and EPA + DHA. It is important to realize that this study was performed in a generally healthy population with moderate to high baseline TG concentrations and elevated LDL-C concentrations. Hence, effect sizes may differ in populations with other baseline lipid values. A limitation of this study is that we did not collect dietary intake data prior and during the study. Though randomization of the subjects to treatment should have removed potential confounding by the background diet, we cannot exclude that other differences in dietary intake confounded the results. Another limitation is that this study was not designed to distinguish the effects of EPA/DHA and PS on TG-lowering. Furthermore, as discussed above, we cannot exclude that small differences in the fatty acid compositions of the placebo and intervention spreads have affected blood lipid concentrations.

In conclusion, the regular intake of a low-fat spread with 2.0 g/day PS and 1.0 g/day EPA/DHA over 4 weeks led to a significant and clinically relevant dual blood lipid benefit based on a decrease of 10.6% in TG and 5.2% in LDL-C, in healthy individuals with modestly elevated blood TG and LDL-C concentrations. This dietary approach could therefore contribute as part of a healthy diet and lifestyle to maintaining low TG and LDL-C concentrations and so help manage the risk of developing CVD.

## Electronic supplementary material

Below is the link to the electronic supplementary material.

Supplementary material 1 (PDF 332 KB)

Supplementary material 2 (PDF 930 KB)
