# Supplementary material for: A low-fat spread with added plant sterols and fish omega-3 fatty acids lowers serum triglyceride and LDL-cholesterol concentrations in individuals with modest hypercholesterolaemia and hypertriglyceridaemia
Source: Eur J Nutr. 2018 May 3;58(4):1615–24. doi: 10.1007/s00394-018-1706-1 (PMC6561982; doi:10.1007/s00394-018-1706-1)
Supplement: Supplementary file 1 — Supplementary material 1 (PDF 332 KB) [file 394_2018_1706_MOESM1_ESM.pdf]

European Journal of Nutrition

A low-fat spread with added plant sterols and fish omega-3 fatty acids lowers serum triglyceride and LDL-cholesterol concentrations in individuals with modest hypercholesterolaemia and hypertriglyceridaemia

Wendy A.M. Blom*, Wieneke P. Koppenol, Harry Hiemstra, Tatjana Stojakovic, Hubert Scharnagl, Elke A. Trautwein

*Corresponding author: Unilever Research and Development Vlaardingen, The Netherlands, Email: wendy.blom@unilever.com

Online Resource I: Effects of placebo and intervention treatments on blood lipid and apolipoprotein measures in the Per Protocol population[a]

Outcome parameter	Placebo group End-of-intervention (LS means + 95% CI)	Intervention group End-of-intervention (LS means + 95% CI)	Absolute difference in LSMeans vs Placebo (95%CI)	Relative difference in LSMeans vs Placebo (95%CI)	P-value
	mmol/L	mmol/L	mmol/L	%	
TG (mmol/L)	1.86 (1.78 to 1.95)	1.66 (1.59 to 1.74)	-0.20 (-0.31 to -0.09)	-10.8 (-16.2 to -5.2)	<0.0001
LDL-C (mmol/L)	4.23 (4.14 to 4.32)	3.99 (3.91 to 4.08)	-0.24 (-0.35 to -0.12)	-5.6 (-8.3 to -2.8)	<0.0001
TC (mmol/L)	6.05 (5.95 to 6.16)	5.80 (5.70 to 5.90)	-0.25 (-0.39 to -0.11)	-4.2 (-6.5 to -1.8)	0.001
HDL-C (mmol/L)	1.24 (1.22 to 1.26)	1.26 (1.24 to 1.28)	0.02 (-0.01 to 0.04)	1.3 (-0.8 to 3.3)	0.225
Non-HDL-C (mmol/L)	4.78 (4.69 to 4.88)	4.51 (4.42 to 4.60)	-0.28 (-0.41 to -0.14)	-5.8 (-8.5 to -3.1)	<0.0001
Remnant-C (mmol/L)	0.54 (0.52-0.56)	0.49 (0.48-0.51)	-0.04 (-0.07 to -0.02)	-8.2 (-3.4 to -12.7)	0.001
ApoAI (mg/dL)	135.1 (133.4 to 136.8)	135.0 (133.3-136.7)	-0.15 (-2.58 to 2.28)	-0.1 (-1.9 to 1.7)	0.904
ApoAII (mg/dL)	40.0 (39.2 to 40.8)	39.0 (38.2 to 39.7)	-1.07 (-2.16 to 0.03)	-2.7 (-5.3 to 0.1)	0.056
ApoCII (mg/dL)	5.4 (5.2 to 5.6)	5.2 (5.0 to 5.4)	-0.19 (-0.45 to 0.07)	-3.6 (-8.2 to 1.2)	0.142
ApoCIII (mg/dL)	14.0 (13.5 to 14.5)	12.9 (12.4 to 13.3)	-1.12 (-1.79 to -0.45)	-8.0 (-12.4 to -3.3)	0.001
ApoE (mg/dL)	12.2 (11.9 to 12.5)	12.1 (11.8 to 12.4)	-0.03 (-0.44 to 0.39)	-0.2 (-3.6 to 3.3)	0.905
ApoB (mg/dL)	100.0 (97.9 to 102.0)	96.3 (94.4 to 98.3)	-3.63 (-6.45 to -0.80)	-3.6 (-6.4 to -0.8)	0.012
ApoB/ApoAI (mg/dL)	0.74 (0.73 to 0.76)	0.72 (0.70 to 0.73)	-0.03 (-0.05 to -0.01)	-3.5 (-6.3 to -0.6)	0.018

[a] *TG triglyceride; LDL-C low-density lipoprotein cholesterol; HDL-C high-density lipoprotein cholesterol; TC total cholesterol; apo apolipoprotein*
