# Supplementary material for: A low-fat spread with added plant sterols and fish omega-3 fatty acids lowers serum triglyceride and LDL-cholesterol concentrations in individuals with modest hypercholesterolaemia and hypertriglyceridaemia
Source: Eur J Nutr. 2018 May 3;58(4):1615–24. doi: 10.1007/s00394-018-1706-1 (PMC6561982; doi:10.1007/s00394-018-1706-1)
Supplement: Supplementary file 2 — Supplementary material 2 (PDF 930 KB) [file 394_2018_1706_MOESM2_ESM.pdf]

European Journal of Nutrition

A low-fat spread with added plant sterols and fish omega-3 fatty acids lowers serum triglyceride and LDL-cholesterol concentrations in individuals with modest hypercholesterolaemia and hypertriglyceridaemia

Wendy A.M. Blom*, Wieneke P. Koppenol, Harry Hiemstra, Tatjana Stojakovic, Hubert Scharnagl, Elke A. Trautwein

*Corresponding author: Unilever Research and Development Vlaardingen, The Netherlands, Email: wendy.blom@unilever.com

Online Resource II Study flow chart

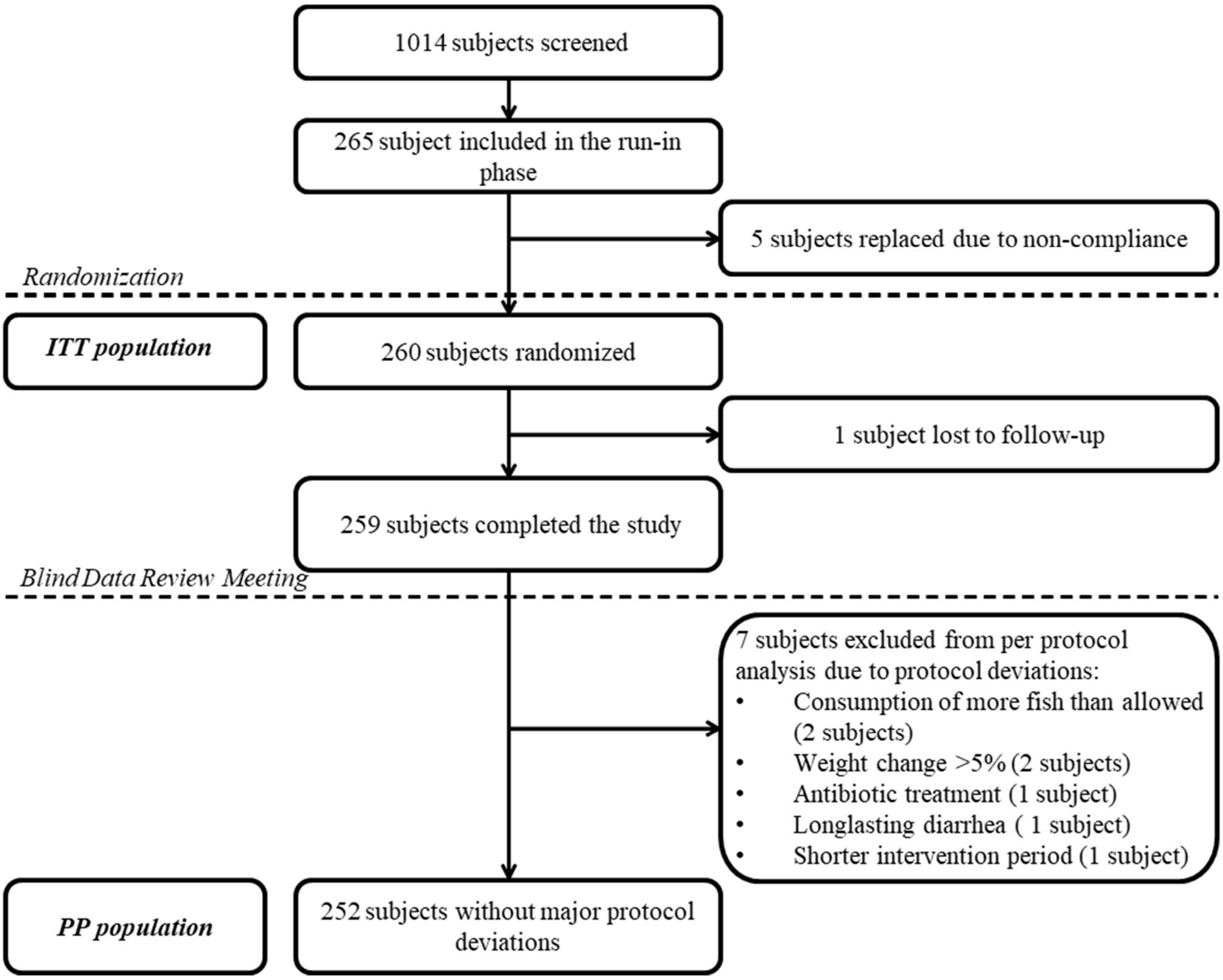

PP: Per Protocol; ITT: Intent To Treat
